# Rapid recombinant protein production from pools of transposon-generated CHO cells

**DOI:** 10.1186/1753-6561-5-S8-P34

**Published:** 2011-11-22

**Authors:** Mattia Matasci, Virginie Bachmann, Lucia Baldi, David L Hacker, Maria De Jesus, Florian M Wurm

**Affiliations:** 1 Laboratory of Cellular Biotechnology, Faculty of Life Sciences, Ecole Polytechnique Fédérale de Lausanne, CH-1015 Lausanne, Switzerland; 2 ExcellGene SA, CH-1870 Monthey, Switzerland

## 

Transient gene expression (TGE) is the most commonly used technology for the rapid production of moderate quantities of recombinant proteins for preclinical studies or for analytical assay development [1,2]. Whereas TGE can provide milligram to gram amounts of recombinant proteins within a time period as short as few days, technical limitations and high costs of plasmid DNA hamper the application of this technology at larger scales. Here we describe an alternative method for the fast production of recombinant proteins from pools of mammalian cells stably transfected with the PiggyBac (PB) transposon. Expression from stably integrated transgene is highly dependent upon the chromatin structure surrounding the site of integration [3]. As a consequence stable cell populations generated by conventional transfection techniques generally show low productivity and reduced expression stability. Several studies showed that the PB transposase mediated transgene insertion predominantly into actively transcribed regions of the mammalian genome [4,5], moreover we recently demonstrated that PB transposition generates high-producing CHO cell lines at a higher frequency than conventional plasmid transfection [6]. These results prompted us to develop a technology based on the use of stable pools generated by PB transposition for the rapid and scalable production of recombinant proteins.

Initial experiments were aimed at determining the proportion of transgene expressing cells as well as the level and stability of recombinant protein production in PB transposed pools. First, we analyzed transgene expression in 952 cell lines recovered from a pool of CHO cells generated by PB-transposition to express tumor necrosis factor receptor as an Fc fusion protein (TNFR:Fc). The cell lines were analyzed by ELISA for TNFR:Fc expression in 4-day batch cultures. The clones showed levels of TNFR:Fc expression up to 360 mg/L with an overall mean of 96 +/- 54 mg/L. PB-transposition resulted in a high percentage (more than 98%) of TNFR:Fc expressing clones (data not shown). Analyses on the stability of transgene expression over time were conducted using a bicistronic PB-donor plasmid allowing co-expression of TNFR:Fc and the enhanced green fluorescent protein (eGFP). Five independent pools of cells were generated by transposition and recovered after 10 days of selection in puromycin. The pools were cultivated for 3 months in the absence of selection and eGFP expression was monitored periodically by flow cytometry. For each pool, the percentage of eGFP-positive cells remained stable over time (Table 1). The stability of transgene expression was further confirmed by TNFR:Fc productivity studies performed in 50-ml cultures at different time points post-transfection. At one month post-transfection, the five pools showed comparable growth and production characteristics, reaching TNFR:Fc titers in the range of 350-500 mg/L in 14-day batch cultures. Similar results were obtained when productivity was tested 2 – 3 months post-transfection (Table 1).

**Table 1:** Analysis of the stability or recombinant protein expression in 5 independent pools of CHO cells.

CHO-Pool	Days post transfection	% eGFP positive cells ^(*)^	TNFR:Fc productivity (mg/L) ^(°)^
	30	92.8 ± 2.1	430 ± 46
**1**	60	93.3 ± 0.2	455 ±10
	90	92.5 ± 1.2	473 ± 10

	30	90.0 ± 0.8	348 ± 36
**2**	60	91.4 ± 0.5	370 ± 8
	90	94.0 ± 1.1	325 ± 35

	30	94.0 ± 1.5	432 ± 25
**3**	60	94.2 ± 1.1	451 ± 28
	90	91.9 ± 1.8	494 ± 16

	30	92.0 ± 0.2	432 ± 30
**4**	60	90.1 ± 2.0	407 ± 23
	90	92.3 ± 1.3	406 ± 28

	30	96.1 ± 1.5	388 ± 52
**5**	60	92.4 ± 1.4	466 ± 19
	90	93.6 ± 0.2	372 ± 12

(*) determined by GUAVA flow cytometry; (°) determined by ELISA.

**Figure 1:**
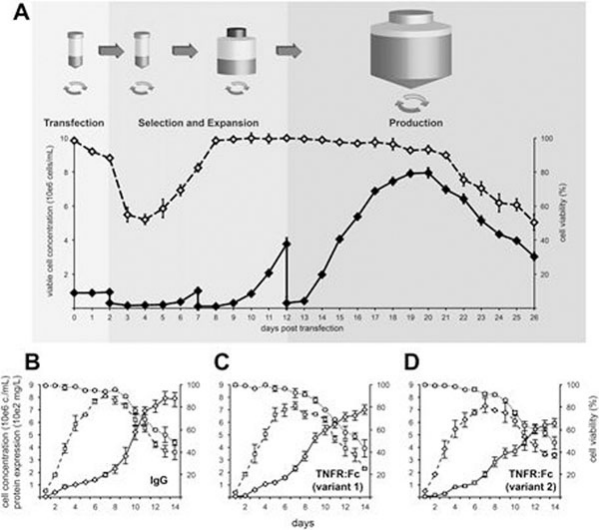
(A) Schematic representation of the protocol for the rapid production of recombinant proteins from pools of transposed cells. This protocol was successfully used to produce a recombinant monoclonal antibody (B) and two variants of TNFR:Fc (C and D). For each bioprocess shown, the percentage of viable cells (dotted lines) the viable cell density (dashed lines), and the recombinant protein titer (solid lines) were measured at the times indicated

We developed a protocol for protein production from transposed cell pools at the 0.5-L scale (Figure 1A). Starting at 2 d post-transfection cells were subjected to 10 days of puromycin selection during which cells were expanded from TubeSpin^®^ Bioreactor 50 tubes into orbitally shaken 250-mL cylindrical bottles. The batch bioprocess was finally started at 12 d post-transfection using orbitally shaken TubeSpin^®^ Bioreactor 600 tubes. Using stable cell pools expressing either an IgG antibody (Fig. 1B) or two TNFR:Fc variants (Fig. 1C, D), we produced 500-750 mg of recombinant protein within a month after transfection.

Our results demonstrated an improved level and stability of transgene expression in transposed pools, indicating usefulness of PB transposed cell pools as a valuable alternative to TGE for the rapid production of recombinant proteins.
